# «If you give them your little finger, they’ll tear off your entire arm»: losing trust in biobank research

**DOI:** 10.1007/s11019-020-09969-w

**Published:** 2020-07-30

**Authors:** Lars Ursin, Borgunn Ytterhus, Erik Christensen, John-Arne Skolbekken

**Affiliations:** 1 Department of Philosophy and Religious Studies, NTNU, 7491 Trondheim, Norway; 2 Department of Public Health and Nursing, NTNU, 7491 Trondheim, Norway; 3 Faculty of Social Sciences, Nord University, 8049 Bodø, Norway

**Keywords:** Consent withdrawal, Confidence, Informed consent, Ethics, Alienation

## Abstract

Why do some people withdraw from biobank studies? To our knowledge, very few studies have been done on the reflections of biobank ex-participants. In this article, we report from such a study. 16 years ago, we did focus group interviews with biobank participants and ex-participants. We found that the two groups interestingly shared worries concerning the risks involved in possible novel uses of their biobank material, even though they drew opposite conclusions from their worries. Revisiting these interviews today reveals a remarkable relevance to present concerns, since the *possible* developments that worried ex-participants and participants 16 years ago now are becoming *realities*. Drawing on conceptual distinctions by sociologist and philosopher Niklas Luhmann, we argue that while ex-participants express a loss of *trust* in the biobank institution to manage the use of their biobank material in a legitimate way, remaining participants expressed *confidence* in the management of the biobank institution to secure their interests. This analysis brings out important aspects of emerging trends in biobank research participation.

## Introduction

Research biobanks are dependent upon donations of blood and other biological samples to pursue their research ambitions. Non-participation has thus been seen as a potential threat to their activity. Consequently, there has been a great interest in individuals’ factual and hypothetical attitudes and reflections on participation in biobank research. Over the past decades this has resulted in an extensive number of studies with consent, sharing of data, and return of results among its most frequently covered topics (for reviewed evidence, see: D’Abramo et al. 2015; Domaradzki and Pawlikowski 2019; Garrison et al. 2016; Husedzinovic et al. 2015). Individuals’ reasons for non-participation in biobank research might to some extent be inferred indirectly from such studies.

When it comes to the perspectives of people who have chosen not to participate in such research, our knowledge is more limited. Part of what we know is that historical experiences and cultural beliefs of various ethnic groups make recruitment for biobank research on individual terms challenging in certain populations (Aramoana and Koea 2020; Kowal et al. 2015; Nooruddin et al. 2020; Sanderson et al. 2017; Lee et al. 2019).

Furthermore, there is evidence that potential participants sometimes have practical reasons for declining participation in biobank research, as shown when participation represents an intrusion in the lives of cardiac care patients (Williams et al. 2013) or when individuals choose not to participate because they are too busy and not willing to give priority to research participation (Ridgeway et al. 2013; Helgesson et al. 2009). Some of the latter individuals were also concerned about confidentiality and privacy issues, as were those individuals who actively refused to participate (Ridgeway et al. 2013). Among people refusing to consent to biobank research in Sweden more than a decade ago, lack of personal gain, genetic privacy, as well as perceived lack of time was the major concern (Melas et al. 2010).

The above description covers the main known reasons for people to choose not to opt *into* biobank research. Opting *out of* such research is, however, another matter. To our knowledge, the results from only one such study is reported in the scientific literature, based on a very limited number of people withdrawing from biobank research (Broekstra et al. 2019).

In this article, we report from a study including persons opting out of biobank research. In our study, we interviewed both participants and ex-participants in the Nord-Trøndelag Health Survey (HUNT). In the county of Nord-Trøndelag in Norway, there has from 1985 until 2019 been four extensive rounds of recruitment to HUNT: HUNT1–HUNT4. In HUNT2, comprising of 65.291 participants recruited in 1995–1997, blood samples were taken and stored in the newly established HUNT Biobank. In subsequent years, amidst the development of and attention to genetics epitomised by the Human Genome Project, the opportunity arose for using the HUNT blood samples for genetic research.

To make use of these samples for genetic research was not straightforward, as there were disputes on whether the samples contained an explicit consent for genetic research (See Skolbekken et al. 2005 for details). This led HUNT to send a letter to the participants in 2002, informing them about the possibility of genetics research on their blood samples, and reminding them about their right to withdraw from the study. Subsequently, participants gave their consent passively by not responding to the letter, whilst those withdrawing were asked for an active response. As a result, 1.187 (1.9%) participants chose to withdraw their samples from the biobank (Holmen et al. 2004).

Previously, we have reported from focus groups with participants who stayed in HUNT (Skolbekken et al. 2005). Here, we will also report from focus groups with HUNT2 participants who decided to withdraw their consent as participants in the biobank part of HUNT, following the 2002 round of information letters. The interviews show concerns regarding a range of issues in the setup and execution of biobank research, as we will display in the findings section. In the discussion section, we will analyse these findings with a special emphasis on the central issue of trust and confidentiality.

The interviews were completed 16 years ago, in 2004. It is natural to ask whether these interviews are still relevant today. Our answer is that they are *even more* relevant, as the reflections of these participants come across as amazingly fresh and timely, engaging directly with the current debate in biobank ethics. More so than they did 16 years ago.

At the time the group discussions took place, the views and conclusions of the consent ex-participants seemed a bit far-fetched. How to deal with the use of samples for problematic genetic research or by contested commercial enterprises were discussed, but in the form of hypothetical future scenarios or ‘exotic’ stories from abroad. The worries remained abstract and academic, as no real-world examples of challenging biobank practices were taking place locally. That is one reason why we put the transcripts from the interviews in a drawer (we will return to additional reasons in the methods section).

It has taken a long while for biobanks to position themselves and get ethically, economically, technically and juridically ready to take on the kinds of projects and collaborations that were discussed as future challenges for biobank participants in the interviews. By now, however, biobank research is maturing in these respects. The relationship between epidemiological and clinical research is much closer, making the relationship between biobank institutions and participants more reciprocal than before.

Currently, discussions are not only restricted to consent issues, but also to worldwide data sharing, huge public–private partnerships within the business of sequencing participants, new policies for returning results, the introduction of new type of recruitment strategies like recruitment by genotypes, and not to forget, the question of storing large amounts of gene data in clouds within or outside our own jurisdiction. This development makes the concerns of our 2004 interview participants more relevant today than at the time they were expressed. Merely hypothetical challenges have become acutely real.

In this article, we will first present and briefly analyse the findings from our interviews with participants and ex-participants. The centrals findings in the interviews are the different views but shared emphasis of participants and ex-participants on the issue of *trust*. We subsequently use these findings as a point of departure to discuss the historical and possible future transformation of trust relations in biobank research participation. This allows us to discuss the issue of trust-based biobank participation in some detail. The benefit of hindsight and opportunity of a longitudinal perspective on the status of biobanking further allows us to distinguish between radically different relations of trust in biobanking.

The interviewees in our study are all very concerned with their relation of trust with the HUNT biobank institution. Some theories of trust just allow trust relations between individuals, not between individuals and social institutions (Offe 1999; Hardin 2002). In this article, our empirical material led us to find a theoretical approach that allows a nuanced view of how and when trust in institutions is possible and rational (Broekstra et al. 2019; Kraft et al. 2018). Thus, we take departure from Niklas Luhmann’s analyses of the relations of trust in social systems (Luhmann 1979, 2000).

Luhmann turned traditional system theories upside down from being dominated by functionalism to becoming communicative social systems characterized by complexity and flexibility (Luhmann 1979; Holmström 2005, 2007). According to Luhmann, traditional system theories describe rational interaction between individuals and social systems as *confidence*- rather than *trust*-based. Confidence refers to knowledge and familiarity: the fact that you know what to expect from others in a social situation (Luhmann 2000; Seligman 1998, p. 39). Complex and flexible social systems are, however, interdependent of each other and not fully predictable for their clients. To have confidence in an institution as such is therefore impossible or irrational. It is just possible and rational to trust *individuals* in systems.

Luhmanns’ system theory opens the possibility of trust in institutions based on *institutionalized mistrust* within the institutions in question, practiced through strict routines of internal control (Offe 1999; Grimen 2009). The trusting client does not need to know and have confidence in individuals in the institution but can rationally trust the institution based on abstract trust in the institutional checks and balances (we illustrate and discuss this in more detail in the discussion section).

In this article, we use the distinction between confidence-based and trust-based participation (Luhmann 2000; Seligman 1998) to analyse our empirical data in light of our research question: Why do some people withdraw from biobank studies? Based on this analysis, we suggest that a move from confidence-based to trust-based participation is crucial to the understand the changes in the “contract” between biobank institutions and participants in the last two decades. We conclude that if current biobanks are to base their participation on trust rather than confidence, they need to make participants able to take on increased responsibility through increased interaction and transparency.

## Study design

In 2004, we invited (1) participants who had consented to genetic research on their samples in the HUNT Biobank, (2) former participants who had withdrawn their previous consent, with the promise of the physical destruction of their samples, and (3) researchers who had an interest in biobank research to participate in a focus group study. Each focus group consisted exclusively of one of these three kinds of participants; there were no mixed groups. In total, we conducted five focus groups with HUNT participants, two focus groups and one individual interview with the former biobank participants (‘ex-participants’), as well as two focus groups and two individual interviews with researchers. The findings reported here are based on the data from the interviews with ex-participants, supplemented with some findings from the groups of participants.

Participants in our study were recruited based on an information letter distributed by HUNT Research Centre. This ensured that we did not know their identity before they made their choice to contact us, and that HUNT did not know which of their participants had chosen to take part in our study. The recruitment procedure was approved by the Regional ethics committee for medical and health research ethics, as part of their approval of the overall study.

Whereas recruitment of the participants was quite easy, recruitment of ex-participants proved to be extremely difficult. [Broekstra et al. (2019) report the same difficulty in recruiting ex-participants]. We contacted 573 HUNT participants that were listed as ex-participants, i.e. close to half of them, and the half that were living in or around the urban areas of the county. Recruitment from rural areas with few potential participants scattered over several municipalities and long distances was not seen as feasible, given the focus group design. Only eight agreed to take part in and showed up for our study. Of the eight ex-participants, two men and two women took part in the first focus group, and two men and one woman took part in the second focus group. One man later took part in an individual interview. Another eight had confirmed to participate in a third focus group, but never turned up on the designated night. This experience left the impression of a group that was disengaged and ‘hard to get’, which was quite discouraging, contributing to our decision to put this part of our data set in the drawer.

In the decades since its establishment the HUNT biobank has gained more participants than it has lost. Since HUNT2, very few HUNT participants have withdrawn their consent to become ex-participants. HUNT databank has registered only 42 withdrawals from HUNT3 (recruitment period 2006–2008) and 126 withdrawals from HUNT4 (recruitment period 2017–2019).

A notable fact of HUNT3 recruitment was that most of the ex-participants from HUNT2 opted into biobank research again by donating new biological samples to the HUNT biobank as part of enrolling. There are strong indications that their initial withdrawal was the outcome of a misunderstanding of the passive consent procedure chosen for the confirmation of the HUNT2 consent on genetic research. This misunderstanding might also contribute to explain why it was so hard for us to recruit ex-participants to our focus groups.

The discussion guide topics were the same for all focus groups, but the questions were altered in accordance with the participants’ choice regarding biobank participation. Both the focus groups and the individual interview were semi-structured, based on the main questions listed in Box 1.

### Box 1: Focus group interview questions with biobank ex-participants/participants


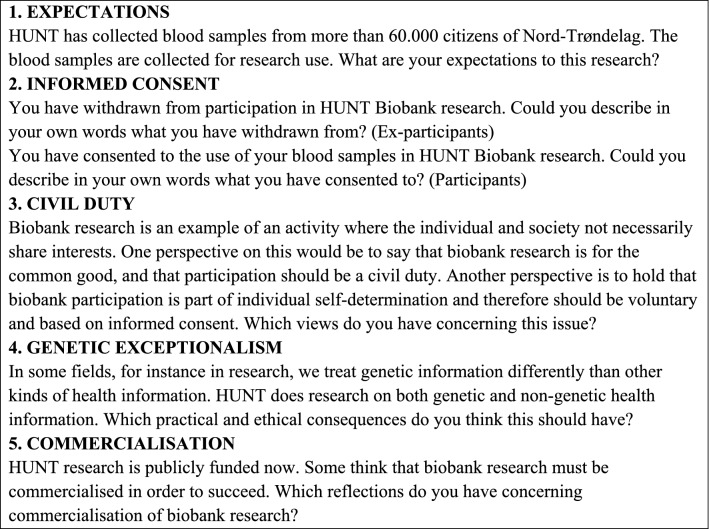


The focus groups were held during two evenings in meeting rooms in public buildings in two urban municipalities. JAS and BY served as moderator and co-moderator of both groups, and LU and EC as research assistants in one group each. The topics were introduced one at a time and presented as written handouts for the participants to read. Discussions lasted for approximately 90 min. In addition, one individual interview with a participant working in Trondheim was completed with JAS as interviewer in JAS’s university office.

All interviews were audiotaped on minidiscs and subsequently transcribed into full text. Data were analysed by the steps of meaning condensation (Kvale 1996) for each interview: all four authors (1) read through all the interviews, (2) identified units of meaning, (3) made comparisons of these units across the interviews to identify common themes, and (4) developed themes into the story line of this article. The interviews were in Norwegian, and all quotes from the interviews were translated by LU in a manner that reflects the actual wording used by the participants.

## Ethical considerations

Research participants have the fundamental right to withdraw from a study at any time and with no questions asked. In this study, we interfered with this right by asking questions. The purpose of the study was to investigate participants’ reasons for withdrawal and to continue to do research on participants that have withdrawn their consent to further research. Consequently, we have some serious ethical issues to address.

Our ethical justification for doing this study despite these challenges is simply that our research group was completely independent of the HUNT-study at the time. Our potential participants were thus not asked to state reasons for their withdrawal to HUNT researchers, but to take part in an independent research project with independent interests and aims. In this way we avoided any kind of unethical psychological pressure on former biobank participants in the sense that they felt they had to justify their withdrawal towards HUNT.

Arguably, the invitation to take part in our study increased rather than restricted their chance to reason and act freely, by offering an opportunity to voice and reflect on their concerns in a project aimed to understand the views of biobank participants and ex-participants. The views of biobank ex-participants are very seldom heard in the ethics literature, which is a serious loss and possible bias that led us to include this group in the study and eventually write this article.

## Findings

In this section, we present our findings structured by the seven major concerns that we have identified in the interviews with ex-participants. We have supplemented this material with some quotes from the interviews with participants, in order to make some illustrative comparisons between the groups. The seven major concerns voiced were (1) personal motivations, (2) justice, (3) surveillance, (4) commercialization, (5) data sharing, (6) broad consent, (7) involvement and trust. These concerns were not expressed in unison by the discussants but represent concerns that were introduced in the group discussions by one or more of the participants.

### Personal motivations

Scepticism about the drivers of biobank research was voiced by the ex-participants. They suspected data collection and research driven by illegitimate personal motivations, unjust policies, surveillance possibilities, and objectionable commercial goals. We will return to the last three drivers immediately below.

Concerning the first driver, ex-participants strongly doubted that the aim of doing epidemiological research in order to prevent major lethal diseases express the real motivation of HUNT researchers, as stated by HUNT in their information leaflets. This aim might express a lofty ideal, but it was seen as one that is a bit naïve in terms of explaining what gets biobank researchers up in the morning. Referring to their own work experience, they found that it was much more likely to be researchers furthering their own career.

Interviewee 1: Many diseases aren’t researched by the doctors because there is more money and prestige in cancer research and the like. Diseases that are hard to find a cure for are not so interesting because it takes too long time for doctors to get funding and win prizes. (2nd focus group of ex-participants)

The ex-participants presumed that in their everyday reality, researchers had to pursue quick results and career promotion—otherwise they would soon be out of business.

### Justice

Injustice is another aspect of questionable research motivation, this time at a political level. It was portrayed as unjust to prioritize the funding of biobank endeavours like HUNT, set to do research on the health problems primarily affecting an affluent Norwegian population, instead of doing research on the health problems of poor people in other parts of the world.

Interviewee 2: I think it’s unfair that we use all this money in the Western world on diseases of the rich, while no-one cares about the diseases that billions of poor people have. (2nd focus group of ex-participants)

For ex-participants, the use of resources on biobank research had to be justified not only ethically but also politically. While taking part in HUNT research might not be problematic in a narrow research ethical perspective dealing mainly with participant risks, it was seen to be problematic in a broader global ethico-political perspective of priority setting.

### Surveillance

Privacy protection and increasing possibilities of surveillance in general were other major concerns among the ex-participants. The tracking of physical and virtual movements via mobile phones were among the topics leading to scepticism. Collection of blood samples and genetic data in biobank repositories were read into the narrative of enabling more comprehensive surveillance by wider collection and collation of data produced by novel technologies.

Interviewee 1: We are heading towards the society George Orwell described in “1984”. Small steps are taken that will lead us to something dangerous. A bit here and a bit there and suddenly we’re there – not good. (2nd focus group of ex-participants)

HUNT was not seen as aiming for any kind of undue surveillance at all, but no-one could guarantee against surveillance and forensic analyses of the biobank material by unknown governments in the future.

### Commercialisation

Among the drivers that the ex-participants were sceptical of, commercial interest as driver of biobank research was nevertheless voiced as by far the most problematic. The perceived aims and interests of commercial interest and actors were both (1) amplifying the other problematic aspects of the biobank mentioned above, and (2) adding a highly problematic aspect of making economic profit from the public endeavour that the ex-participants initially had signed up for. Ex-participants are willing to participate in research for the common good, but not when it serves the interests of commercial companies.

Interviewee 1: If the research is funded by public money, not by commercial companies, I’m in. (2nd focus group of ex-participants)

With the introduction of commercial interests, other aims than doing and contributing to the common good are introduced. This was seen as opening up diversion of medical resources to benefit the rich rather than the poor, bending ethical norms to enter into problematic research fields like genetic modification and the like, and using genetic information for discriminatory practices like refusing health insurance to the genetically disadvantaged. The ex-participants did not think that the HUNT organization was able to handle the interests of commercial companies in a good way and secure the interest of HUNT participants to contribute without privacy risks to medical research just for the common good.

When I am asked to take part in something, and they are unable to make clear the role of commercial interests, well it does not increase the sum of money on their trust fund, so to say. (Individual interview, ex-participant)

The outcome of the ex-participants’ risk assessments differed from the conclusion drawn by the participants, even if the same worries were expressed by participants in strikingly similar terms as the worries regarding questionable drivers stated above by ex-participants.

Interviewee 1: I’m afraid that big foreign companies get access, and you know what... including cloning and genetic modification and all that... I’m against that. That will be misuse, in my eyes. (2nd focus group of participants)

Interviewee 4: Universities nowadays are expected to attract commercial funding and finance their own business, and the same thing might happen to the biobank. This means that the owners of the biobank are no longer concerned with public health but with making profits...

Interviewee 3:… and insurance companies are interested in such data.

Interviewee 4: Yes, then biobank research is no longer for the people but against the people. (1st focus group of participants)

The prospect of commercial access to and use of biobank material was a major concern in both groups. The difference between these groups was mainly in their assessments of the confidence in and power of HUNT to make any commercial involvement comply with the stated aims of the HUNT study. While ex-participants strongly doubted the ability of HUNT to keep disruptive commercial forces at bay, participants were confident that HUNT would know where to draw the line to protect the aim of research for the common good and to protect participants from risks and undue use of their biobank material.

### Data sharing

The prospect of data sharing caused concern among ex-participants, especially because of the lack of information and clarity regarding the nature and scope of such sharing of data. Sharing of data with commercial companies again raised most alarm, because of the heterogeneity of aims between public and commercial medical research: for the public good or for shareholder profit.

The prospect of sharing data in general meant an erosion of participant control over the use of one’s own biological data, and thus the question became one of confidence in HUNT to control the use of data according to the interests of the participants. The ex-participants again doubted the ability of HUNT to be good stewards of the interests of HUNT participants.

Interviewee 1: I’m okay with research on my blood and genes to find diseases and so on, but that’s not enough, because if you give them your little finger, they’ll tear off your entire arm, and you don’t know where it’s going to stop. That’s the problem. I want to be in control over my blood and my genes. That’s why I do not want to be in such a research database. It’s okay that my doctor and the hospital keep my data. But to have my data shared with all researchers and labs in the world, maybe even patenting something that belongs to me, that’s what I am not okay with, because my blood belongs to me and no-one else. (2nd focus group of ex-participants)

The lack of stated boundaries to data exchange led to a perceived lack of overview and control of the access and use of the biological data in the HUNT Biobank, which put ex-participants in the situation of having to trust HUNT more or less blindly.

### Broad consent

Ex-participants expressed strong reservations concerning the appropriateness of the use of broad consent to enrol biobank participants in HUNT. The lack of information regarding the (limits of) use and sharing of their data effectively made broad consent a blanket consent in their eyes. The information provided was too abstract and general, and the failure of the researchers to clearly state what kind of research projects the biological data would (and would not) be used for, made the ex-participants feel that leaving their biological data in the HUNT biobank was too risky.

Interviewee 4: I would have agreed to take part in many kinds of research for the benefit of future generations and myself. However, when I realized that the researchers did not know what kind of research they would use the samples for, I withdrew my consent. (1st focus group of ex-participants)

They ask for a blanket consent to any future kind of research, for any kind of commercial company. Such a blanket consent is too broad. A more specified [periodic] consent would have increased my trust. I haven’t given them my blood, they’ve just got a licence. (Individual interview, ex-participant)

Interestingly, while ex-participants and participants had quite similar assessments of what would be problematic impacts of commercial interests entering the HUNT Biobank domain, their assessments of the use of broad consent were by contrast quite dissimilar. Participants did not feel that broad consent made participation too risky, or that it was unclear what kind of research their data would be part of. They had confidence in HUNT researchers to do research—also with researchers from other research units—in line with the stated aims of the HUNT study.

Interviewee 5: Concerning consent, I place my trust in health research and believe that they will use my blood sample in the right way and not misuse it. And use it for serious research. I’ve no objections to that, so I’ve given my consent to that, and I’m confident that they will do that. (3rd focus group of participants)

The confidence expressed by participants was not echoed by ex-participants, who felt that they were asked to place too much trust in HUNT researchers based on too little information and delimitation. Ex-participants consequently argued for much more specific consent requirements to biobank research.

### Involvement and trust

Even though they were rather hard pressed to come up with any kind of detailed description of what they had consented to, the participants did not express any lack of information from HUNT. They were, moreover, not worried about their lack of understanding of the details of their involvement. The crucial point for them was their confidence in HUNT to use their samples for uncontroversial medical research aimed to benefit future generations. Exactly how this would be done, they did not know, and did not care to know, because it did not really matter. The biobank institution had the responsibility anyway, and the participants had confidence in HUNT biobank.

Interviewee 2: Biobanks, well, I don’t know what kind of research that’s going on there. This is all based on trust on my part. In my mind, I have made myself or parts of myself available for research. You might think that I’m naive or whatever, but I think that we must dare something to make progress. (1st focus group of participants)

For the ex-participants, ignorance did matter. Once they for various reasons had started to question whether it really was reasonable to have confidence in HUNT, they experienced a lack of participant information and participant involvement that in the end was felt as a lack of respect.

Interviewee 1: We don’t know what they are looking for. In other parts of society there is an emphasis on participant involvement, on research that should be initiated by and led by participants. But here in HUNT, where I feel that *we* are the most important participants and contributors, we have absolutely no influence on the direction of this.. it seems like there is a medical elite that do whatever they want with my DNA. (1st focus group of ex-participants)

For the participants, the HUNT information setup was ok. For the ex-participants, it was not. The setup did not cater for the ex-participants’ need for information.

I sent HUNT a letter and asked for some clarifications of issues I found very unclear. And the answers I got back from HUNT was not at all satisfactory, but arrogant and failed to address my questions. I felt their message was that I’d better shut up because “they knew what they were doing”. (Individual interview, ex-participant)

The concerns regarding transparency and involvement were mostly aired by people with higher education, including participants working as researchers. For these ex-participants, HUNT was not sufficiently equipped to answer basic questions from participants nor to repair even minor cracks in confidence.

After I got the unsatisfactory answers from HUNT, I realised that I had lost my trust in these people, that I no longer trust them to know what they are doing. And that was exactly what they asked me to do: to trust them. (Individual interview, ex-participant)

The information and interaction available did not suffice to answer their questions of HUNT research policies, privacy protection, and trustworthiness. Thus, the ex-participants felt that it became too risky for them to leave their biological material in the biobank—and exited.

## Discussion

Why did ex-participants withdraw, and participants uphold their consent? Some of the concerns of the ex-participants were matters of plain *disagreement* with HUNT policies, or with aspects of the establishment of biobank institutions as such. Especially regarding the issue of justice, withholders expressed their disagreement with funding infrastructures targeted to doing research on well-off westerners instead of dealing with the more basic health problems of developing countries. This cannot by itself explain the withdrawal, however, as these aspects were the same when they consented to take part in the first place.

Broad consent involves some kind of trust, and most of the reflections of our interviewees regarding consent revolved around the concept of trust. This is in line with the results of the recent study by Broekstra et al. (2019). Regardless of whether they belong to the group of participants or ex-participants, they emphasised that HUNT participation is based on trust: trust in HUNT research to provide some sort of public good without risking the health or privacy of the participants (Skolbekken et al. 2005; De Vries et al. 2019).

This relationship of trust was both vague and strict, broad and narrow. It was *vague* regarding the nature of the research and the use of data and samples from participants. Participants had very vague ideas of what kinds of research projects their data went into.

The participants were still quite okay with this and did not mind giving a *broad* consent to all kinds of completely unknown research projects.

There were, however, *strict* borders around this “green zone” of data use. The premise of the green zone confidence of the participants was that HUNT was regarded as strictly (self-) regulated to ensure that the use of biobank material stayed on a *narrow* path of completely uncontroversial research. The internal and external bodies ethically assessing and approving use of HUNT Biobank material was assumed to be very conservative and cautious and allow nothing but low-risk use. Thus, any possible risks or contested use of the material, like giving access to commercial companies or doing genetic research, should be strictly regulated and fully transparent. This was central to the perceived contract between HUNT and the participants.

Given this contract, why did the participants and ex-participants of our study arrive at opposite ways of acting? Their understanding of the contract was the same, their expectations for the benefit of HUNT research was the same, and their concerns and worries concerning risks and exploitation was the same. In what way was the broad consent given by ex-participants narrower than the broad consent given by participants?

Participants and ex-participants united in viewing activities like genetic research and commercial use as ethically and politically challenging (Kraft et al. 2018; De Vries et al. 2019). Ex-participants, however, simply had *more* trouble with these challenges than participants. Ex-participants gave HUNT less room for manoeuvring than participants. In this way, the broad consent to biobank activity was in effect *narrower* when given from ex-participants than from participants.

Luhmann’s distinction between risk versus certainty and risk versus danger brings out an important aspect of the differences in thinking between participants and ex-participants. While risk versus danger denotes the inescapable risks of life, risk versus certainty denotes risks that are due to risk-taking as part of individual and institutional decision-making—and consequently individual and institutional responsibility—characteristic of the *risk society* (Luhmann 1990).

In the HUNT context, this can correspond to the belief in and ability of a system like HUNT to make sure that the system is shielded from outside risks, and that the risks within the system are minimized, versus holding that there is no way to eliminate the inherent risks of biobank participation. The participants approach HUNT as a functionalistic social system with inherent knowledge about risk in their activities and decision-makings. Consequently, HUNT as an institution bear the potential risks and protect the interacting parts for danger. The ex-participants approach HUNT as a flexible communicative social system interacting with other systems and individuals. Consequently, all interacting partners must to bear parts of the responsibility and risks—including the participants.

### Distinguishing between confidence and trust

“Trust-based" biobank participation: what does it mean? A significant feature of biobank participation is its dependence on broad consent. From the early days of biobanking, the use of broad consent has been contested, because it is questionable whether it fulfils the criteria of *informed* consent. The function of broad consent is to make it possible to recruit participants without offering specific information of the research project they will take part in. Instead, they broadly consent to take part in certain *kinds* of research projects. It is then left to ethics committees to assess whether projects are of the kind broadly consented to by the participants.

The reliance on broad consent in biobank research is in this way a reliance on trust. Without trust in the biobank institution and approval authorities to make sure that data are used according to the broad consent, there will be no biobank participants (Boers et al. 2015; Steinsbekk and Solberg 2011).

However, are we sure that the concept of “trust” is the right concept here? In order to understand trust-relations in biobanking better, we will draw some useful conceptual distinctions. Niklas Luhmann’s distinction between “confidence” and “trust” provides the point of departure for this analysis. For Luhmann, *confidence* is to have certain expectations towards other people, while *trust* in addition includes an acknowledgement of taking a risk (Luhmann 2000). Thus, if you have confidence in other people, you will blame *them* if they disappoint you. If you trust someone and they disappoint you, however, you will also blame *yourself* for taking the risk involved in trusting (Luhmann 1979, p. 24). By trusting someone, you know that there is a risk that they will disappoint you, but you deliberately take this risk.

According to Luhmann’s distinction, confidence is further a relation that belongs to the sphere of familiarity, while risk belongs to the sphere of the unfamiliar. This means that “lack of confidence will lead to feelings of alienation” (Luhmann 2000, p. 103), while “lack of trust, on the other hand, simply withdraws activities” (Luhmann 2000, p. 104). In the biobank context, then, this would entail that lack of confidence will lead to a feeling of alienation of and withdrawal by participants, while a breach of trust will lead to withdrawal of biobank activities (we will expand on this point below).

### Participation based on confidence

The still on-going HUNT-study has for four decades had a very strong local profile. Arriving at the HUNT survey field stations, participants have been met by local health care personnel, offering a personal health check in addition to the research data procurement. The HUNT Research Centre is located in the midst of the county of recruitment, and participant information emphasises that HUNT data are locally stored and controlled by HUNT staff living in the county, not by an outside institution prone to exploit the county population’s willingness to take part. The HUNT communication strategy has been to give participants a feeling of closeness and ownership of the HUNT institution: HUNT has been presented as a reason to be proud of the entrepreneurship of local researchers and the altruism of the local community, and a chance for participants to contribute to build local excellence and take part in something bigger than oneself.

In this way, HUNT has aimed to create a relation to participants based on familiarity. Applying Luhmann’s distinction, this should set the scene for participants to have confidence in HUNT, rather than trust. To consent to HUNT research should be a matter of confidence in persons and institutions guided by familiar norms, not to place trust in persons and institutions with unfamiliar norms or controversial activities.

Even though the nature of biobank research is vague and unfamiliar, contributing to HUNT Biobank should be a matter of confidence rather than taking any kind of personal risk. The understanding of the broad consent given by HUNT participants should not be “I place my trust in HUNT”, but “I have confidence in HUNT”. Participants are not asked to take any risk or responsibility, as HUNT (should) guarantee very low risk participation, both concerning risks of security breaches and of illegitimate use of participant information. Shit happens, of course, but within the sphere of confidence, HUNT should take full responsibility for making and assessing the risk for participants to be very low.

This confidence-based biobank participation is vulnerable to alienation. The introduction of unfamiliar elements, in the form of norms, activities, persons or institutions can lead to lack of confidence. Alienation and withdrawal can result from the familiar being unable to familiarize and absorb the unfamiliar. In our study, the participants and ex-participants had parted their ways regarding the familiarity of HUNT Biobank. Reacting to the prospect of genetic research on the biobank material, the ex-participants were alienated (and chose to withdraw), while the participants relied on HUNT to domesticate any future genetic research and guarantee continued participation with very low risk.

### Participation based on confidence versus trust

Biobank participation based on trust is quite different from biobank participation based on confidence. The difference is clearly brought out in our findings, even though our study participants make no conceptual distinction between “trust” and “confidence” in their statements—as the Norwegian word for trust (“tillit”) covers both concepts: while participants express confidence by being quite okay with their broad consent and lack of detailed knowledge of biobank research, ex-participants express alienation.

As an effect of alienation, the relation between HUNT and ex-participants becomes a matter of trust. For the ex-participants, HUNT can no longer take full responsibility for very low risk participation. Therefore, if the ex-participants had chosen to continue their participation, that would have implied for them that they themselves would have had to assess the risk of participation and take part in the responsibility for risk exposure in HUNT. (Of course, some of the participants might have gone along with such trust-based participation, and as shown above in the findings section, some participants hint at that in our interviews.)

The uneasiness with broad consent and the call for more specific consent by the ex-participants is a way to handle such responsibility and reduce risk. In a trust-based relation between biobanks and participants, where the element of risk is present, improved means of control by participants are vital to avoid withdrawal. Consequently, ex-participants demand both increased transparency and careful policy-work by biobank institutions. This is echoed in the findings of Broekstra et al. (2019).

Two concerns were prominent for the confidence/trust of our study participants: commercialisation and genetic research. There is an important difference regarding the relation of responsibility concerning the two: while commercialisation in the main is a matter of collective risk exposure, genetic research is primarily a matter of personal risk exposure. The commercialisation issue is a matter of biobank strategy, and any risk exposure can be fully managed at the policy level.

The risks of genetic research, on the other hand, can only to some extent be managed at the policy level (for instance by blocking access for insurance companies to genetic information from biobanks). If their biobank material is used for genetic research involving potential individual recall by genotype or return of actionable results, it is in the end up to biobank participants to assess the risk of being involved and to take the responsibility for their decisions.

By including ethically challenging practices like public–private partnerships and possibilities of feedback from genetic research, current biobanking might be moving in the direction of being inherently trust-based, while biobanking in the past was not. Biobanking in the days where collaboration between the health industry and genetic research were just hypothetical future possibilities, not actual on-going realities, was based on confidence rather than trust. The biobank institution took the responsibility, not the participant.

That is why, with the benefit of hindsight, we can say that the reflections of the ex-participants in our study are more relevant today than 16 years ago. Being ahead of their time, and considering future risks as current risks, their reflections on important issues of having a trust-based relation with biobanks go directly into current debates on biobank ethics.

The similarity of concerns but difference in conclusions between participants and ex-participants allowed us to highlight an important distinction between biobank participation based on confidence versus trust. If we go from confidence-based to trust-based participation, changes in the ethical and legal set-up of biobanking are required. To make trust-based biobank participation work, biobanks need to make participants able to take on the responsibility they are given. This means increased interaction and transparency.

Interestingly, this is exactly what is happening now. Emerging participant interaction in the form of digital platforms potentially transform the risk and responsibility dimension of biobank participation: inviting the participant to be in control and make choices regarding involvement might also invite the participant to take increased responsibility for the risks involved.

This shift might also—at last—make possible the kind of participant engagement in biobanking that for decades has been called for. Despite many ingenious initiatives, the establishment of participant engagement in biobanking has been like flogging a dead horse (Goisauf and Durnová 2019). Again, with hindsight, the reason for this is in plain view: why should anyone engage in an enterprise where someone else makes every decision and takes full responsibility? In the current situation, however, where there are real choices to be made and real responsibility to be taken by biobank participants, the ground for engagement is much more fertile.

As noted by Luhmann, distrust retracts activities. If biobanks are unable to build trust regarding challenging activities like genetic research and industry collaboration, research projects involving such activities will have to be fully or partly withdrawn. On the other hand, activities that involve individual or collective risk-taking have the potential to create and attract more engaged biobank participants. With trust-based participation, biobanks might win some, and lose some.

Of course, if the biobank can regain full responsibility and guarantee risk-free participation, either by retracting activities or succeed with strategies where familiarity absorbs unfamiliarity, confidence-based participation is again achievable. The downside of such a strategy, however, can be significant a loss of research opportunities and participant engagement.

## Conclusion

In this article, we have discussed findings from our interview study with participants and ex-participants from biobank research. Ex-participants and participants largely had shared concerns regarding biobank participation, but participants were more concerned and therefore had more restrictions on the broad consent they had given. Based on Luhmann’s distinction between confidence and trust, we have traced how traditional confidence-based biobank participation currently might point the way to participation based on trust. This development is characterised by participants taking more risks and responsibility. To make this legitimate, biobanks must implement new demands of transparency and interaction.

Part of the title of this article is “losing trust in biobank research”. From our discussion, we see that the title could as well have been “placing trust in biobank research”. Because that is what the ex-participants (and possibly some participants) in our study did 16 years ago, and biobank institutions might be doing now: changing the relation between biobanks and participants from being a matter of confidence to become a matter of trust.

## References

[CR1] Aramoana Jaclyn, Koea Jonathan (2020). An integrative review of barriers to indigenous peoples’ participation in biobanking and genomic research. JCO Global Oncology.

[CR2] Boers Sarah N, van Delden Johannes J.M., Bredenoord Annelien L (2015). Broad consent is consent for governance. American Journal of Bioethics.

[CR3] Broekstra Reinder, Aris-Meijer Judith, Maeckelberghe Els, Stolk Ronald, Otten Sabine (2019). Trust in centralized large-scale data repository: A qualitative analysis. Journal of Empirical Research on Human Research Ethics.

[CR4] D’Abramo Flavio, Schildmann Jan, Vollmann Jochen (2015). Research participants’ perceptions and views on consent for biobank research: A review of empirical data and ethical analysis. BMC Medical Ethics.

[CR5] De Vries RG, Raymond G, Ryan Kerry A, Gordon Linda, Krenz Chris D, Tomlinson Tom, Jewell Scott, Kim Scott Y. H (2019). Biobanks and the moral concerns of donors: A democratic deliberation. Qualitative Health Research.

[CR6] Domaradzki Jan, Pawlikowski Jakub (2019). Public attitudes toward biobanking of human biological material for research purposes: A literature review. International Journal of Environmental Research and Public Health.

[CR7] Garrison Nanibaa’ A, Sathe Nila A, Matheny Antommaria Armand H, Holm Ingrid A, Sanderson Saskia C, Smith Maureen E, McPheeters Melissa L, Clayton Ellen W (2016). A systematic literature review of individuals’ perspectives on broad consent and data sharing in the United States. Genetics in Medicine.

[CR8] Goisauf Melania, Durnová Anna P (2019). From engaging publics to engaging knowledges: Enacting “appropriateness” in the Austrian biobank infrastructure. Public Understanding of Science.

[CR9] Grimen Harald (2009). Hva er tillit? [What is trust?].

[CR10] Hardin Russell (2002). Trust and trustworthiness.

[CR11] Helgesson Gert, Hansson Mats G, Ludvigsson Johnny, Swartling Ulrica (2009). Practical matters, rather than lack of trust, motivate nono-participation in a long-term cohort trial. Pediatric Diabetes.

[CR12] Jostein Holmen, Kjelsaas May Britt, Krüger Øystein, Ellekjær Hanne, Bratberg Grete, LingaasHolmen Turid, Midthjell Kristian, ArneStavnås Per, Krogstad Steinar (2004). Befolkningens holdninger til genetisk epidemiologi illustrert ved spørsmål om fornyet samtykke til 61.246 personer—Helseundersøkelsen i Nord-Trøndelag (HUNT). Norsk Epidemiologi.

[CR13] Holmström Susanne (2005). Reframing public relations: The evolution of a reflective paradigm for organizational legitimization. Public Relations Review.

[CR14] Holmström Susanne (2007). Niklas Luhmann: contingency, Risk, trust and reflection. Public Relations Review.

[CR15] Husedzinovic Alma, Ose Dominik, Schickhardt Christoph, Fröhling Stefan, Winkler Eva C (2015). Stakeholders’ perspectives on biobank-based genomic research: Systematic review of the literature. European Journal of Human Genetics.

[CR16] Kowal Emma, Greenwood Ashley, McWirther Rebekah E (2015). All in the blood: A review of aboriginal Australians’ cultural beliefs about blood and implications for biospecimen research. Journal of Empirical Research on Human Research Ethics.

[CR17] Kraft Stephanie A, Cho Mildred K, Gillespie Katherine, Halley Meghan, Varsava Nina, Ormond Kelly E, Luft Harold S, Wilfond Benjamin S, Lee Sandra Soo-Jin (2018). Beyond consent: Building trusting relationships with diverse populations in precision medicine research. American Journal of Bioethics.

[CR18] Kvale S (1996). Interviews: An introduction to qualitative research interviewing.

[CR19] Lee Sandrea S.-J., Cho Mildred K, Kraft Stephanie A, Varsava Nina, Gillespie Katie, Ormond Kelly E, Wilfond Benjamin S, Magnus David (2019). “I don’t want to be Henrietta Lacks”: Diverse patient perspectives on donating biospecimens for precision medicine research. Genetics in Medicine.

[CR20] Luhmann Niklas (1979). Trust and power.

[CR21] Luhmann Niklas (1990). Soziologische Aufklärung.

[CR22] Luhmann, Niklas. 2000/1988. Familiarity, confidence, trust: Problems and alternatives. In *Trust: Making and breaking cooperative relations*, ed. Diego Gambetta, 94–107*.* Oxford: Basil Blackwell.

[CR23] Melas Philippe A, Sjöholm Louise K, Forsner Tord, Edhborg Maigun, Juth Niklas, Forsell Yvonne, Lavebratt Catharina (2010). Examining the public refusal to consent to DNA biobanking: Empirical data from a Swedish population-based study. Journal of Medical Ethics.

[CR24] Nooruddin Mohammed, Scherr Courtney, Friedman Paula, Subrahmanyam Ramesh, Banagan Jeff, Moreno Diana, Sathyanarayanan Myurani, Nutescu Edith, Jeyaram Tharani, Harris Mary, Zhang Honghong, Rodriguez Adriana, Shaazuddin Mohammed, Perera Minoli, Tuck Matthew (2020). Why African Americans say “No”: A study of pharmacogenomic research participation. Ethnicity & Disease.

[CR25] Offe Claus, Warren Mark E (1999). How can we trust our fellow citizens?. Democracy and trust.

[CR26] Ridgeway JL, Han LC, Olson JE, Lackore KA, Koenig BA, Beebe TJ, Ziegenfuss JY (2013). Potential bias in the bank: What distinguishes refusers, nonresponders and participants in a clinic-based biobank?. Public Health Genomics.

[CR27] Sanderson Saskia C, Brothers Kyle B, Mercaldo Nathaniel D, Clayton Ellen Wright, MathenyAntommaria Armand H, Aufox Sharon A, Brilliant Murray H, Campos Diego, Carrell David S, Connolly John J. M, Conway Pat, Fullerton Stephanie M, Garrison Nanibaa A, Horowitz Carol R, Jarvik Gail P, Kaufman David, Kitchner Terrie E, Li Rongling, Ludman Evette, McCarty Catherine A, McCormick Jennifer B, McManus Valerie D, Myers Melanie F, Scrol Aaron, Williams Janet L, Shrubsole Martha J, Schildcrout Jonathan S, Smith Maureen E, Holm Ingrid A (2017). Public attitudes toward consent and data sharing in biobank research: A large multi-site experimental survey in the US. American Journal of Human Genetics.

[CR28] Seligman Adam B (1998). Trust and sociability: On the limits of confidence and role expectations. American Journal of Economics and Sociology.

[CR29] Skolbekken JA, Ursin LØ, Solberg B, Christensen E, Ytterhus B (2005). Not worth the paper it's written on? Informed consent and biobank research in a Norwegian context. Critical Public Health.

[CR30] Steinsbekk Kristin Solum, Solberg B Berge (2011). Biobanks-when is re-consent necessary?. Public Health Ethics.

[CR31] Williams Pamela Holtzclaw, Nemeth Lynne S, Sanner Jennifer E, Frazier Lorraine Q (2013). Thematic analysis of cardiac care patients’ explanations for declining contribution to a genomic research-based biobank. American Journal of Critical Care.

